# Secreted phospholipase A_2_ regulates intercellular communications by coordinating extracellular phospholipid metabolism

**DOI:** 10.1093/intimm/dxaf027

**Published:** 2025-05-20

**Authors:** Makoto Murakami

**Affiliations:** Laboratory of Microenvironmental and Metabolic Health Science, Center for Disease Biology and Integrative Medicine, Graduate School of Medicine, The University of Tokyo, 7-3-1 Hongo, Bunkyo-ku, Tokyo 113-8655, Japan

**Keywords:** extracellular vesicle, lipid mediator, lipidomics, metabolism, microbiome

## Abstract

Lipids play fundamental roles in life. In essence, “phospholipase A_2_” (PLA_2_) indicates a group of enzymes that release fatty acids and lysophospholipids by hydrolyzing the *sn*-2 position of glycerophospholipids. To date, more than 50 enzymes that possess PLA_2_ or related lipid-metabolizing activities have been identified in mammals and are subdivided into several families in terms of their structures, catalytic mechanisms, tissue/cellular localizations, and evolutionary relationships. Among the PLA_2_ superfamily, the secreted PLA_2_ (sPLA_2_) family contains 11 isoforms in mammals, each of which has unique substrate specificity and tissue/cellular distributions. Recent studies using gene-manipulated (knockout and/or transgenic) mice for a full set of sPLA_2_s have revealed their diverse roles in immunity, metabolism, and other biological events. Application of mass spectrometric lipidomics to these mice has allowed the identification of target substrates and products of individual sPLA_2_s in tissue microenvironments. In principle, sPLA_2_s hydrolyze extracellular phospholipids such as those in extracellular vesicles, microbes, lipoproteins, surfactants, and ingested foods, as well as phospholipids in the plasma membrane of activated or damaged cells, thereby exacerbating or ameliorating various diseases. The actions of sPLA_2_s are dependent on, or independent of, the generation of free fatty acids, lysophospholipids, or their metabolites (lipid mediators) according to pathophysiological contexts. In this review, I will make an overview of recent understanding of the unexplored immunoregulatory roles of sPLA_2_s and their underlying lipid pathways, especially focusing on their unique actions on extracellular vesicles, activated/damaged cells, and gut microbiota.

## Teaser

How sPLA_2s_ control lipid metabolism in immune cells

## Introduction

Phospholipase A_2_ (PLA_2_) represents a group of lipolytic enzymes that principally hydrolyze the *sn*-2 position of glycerophospholipids (hereafter phospholipids). In mammals, the PLA_2_ superfamily contains >50 enzymes, which are further divided into several families based on their structural and enzymatic characteristics (1–4). PLA_2_ has long been implicated in the production of polyunsaturated fatty acid (PUFA)- or lysophospholipid-derived lipid mediators, which variably regulate intercellular signaling in an autocrine or paracrine fashion (5). It is often illustrated in biochemistry textbooks that a PLA_2_ associated with the cytoplasmic face of cell membrane is activated by extracellular stimuli to release arachidonic acid (AA; ω6 C20:4), which is then converted by downstream enzymes to eicosanoids, including prostaglandins (PGs) and leukotrienes. In most cases, this PLA_2_ refers to group IVA cytosolic PLA_2_ (cPLA_2_α), which is regulated by cytosolic Ca^2+^ and phosphorylation (6, 7). Beyond this central dogma of PLA_2_ research, it has now become obvious that the PLA_2_ superfamily also contributes to other biological events, including membrane phospholipid remodeling, energy production, barrier function, organellar degradation, and so on, independently of lipid mediator production (1–4). Some of the PLA_2_ members catalyze even non-PLA_2_ reactions, such as phospholipase A_1_, lysophospholipase, neutral lipid lipase, and transacylase, further implying their diverse functions (1, 2). These aspects render the understanding of PLA_2_ biology more complex than previously thought.

Within the PLA_2_ superfamily, secreted PLA_2_s (sPLA_2_s), a group of extracellular lipolytic enzymes exhibiting strict *sn*-2 specificity for the substrate phospholipids, have the longest history of PLA_2_ research, beginning in the middle of the last century as major components in snake and bee venoms. Following the purification of two prototypic enzymes structurally related to snake venom sPLA_2_s (groups I and II) from pancreatic juice and rheumatoid arthritis fluid in the late 1980s, now being referred to as sPLA_2_-IB (encoded by *Pla2g1b* in mice) and sPLA_2_-IIA (encoded by *Pla2g2a*), respectively, a total of 11 mammalian sPLA_2_s (10 catalytically active and 1 inactive isoforms) have been identified to date and are structurally subdivided into one classical (group I/II/V/X) and two atypical (groups III and XII) branches (8–10). Afterward, several laboratories including our group have generated and analyzed mice with transgenic overexpression or gene targeting of a full set of these sPLA_2_s. It has become evident that, except for sPLA_2_-IIA that is induced in the circulation during systemic inflammation, individual sPLA_2_s principally participate in lipid mobilization within tissue microenvironments where they are intrinsically expressed (9, 10). However, a fundamental question has remained unanswered for many years—“which membranes do serve as hydrolytic targets of sPLA_2_s *in vivo*?”

On the basis of the latest understanding of sPLA_2_s in the past decade, we herein discuss their roles and mechanistic actions in the context of immunology and related biological events. Importantly, sPLA_2_s behave like “lipolytic cytokines” that can coordinate intercellular communications via hydrolyzing various forms of extracellular phospholipids (9, 10). We put a particular focus on the lipolytic actions of sPLA_2_s on extracellular vesicles (EVs), activated or damaged cells, and microbiota *in vivo*, as evidenced by recent studies using gene-manipulated mice in combination with comprehensive lipidomic analysis.

## General aspects of sPLA_2_s

**Figure 1.**
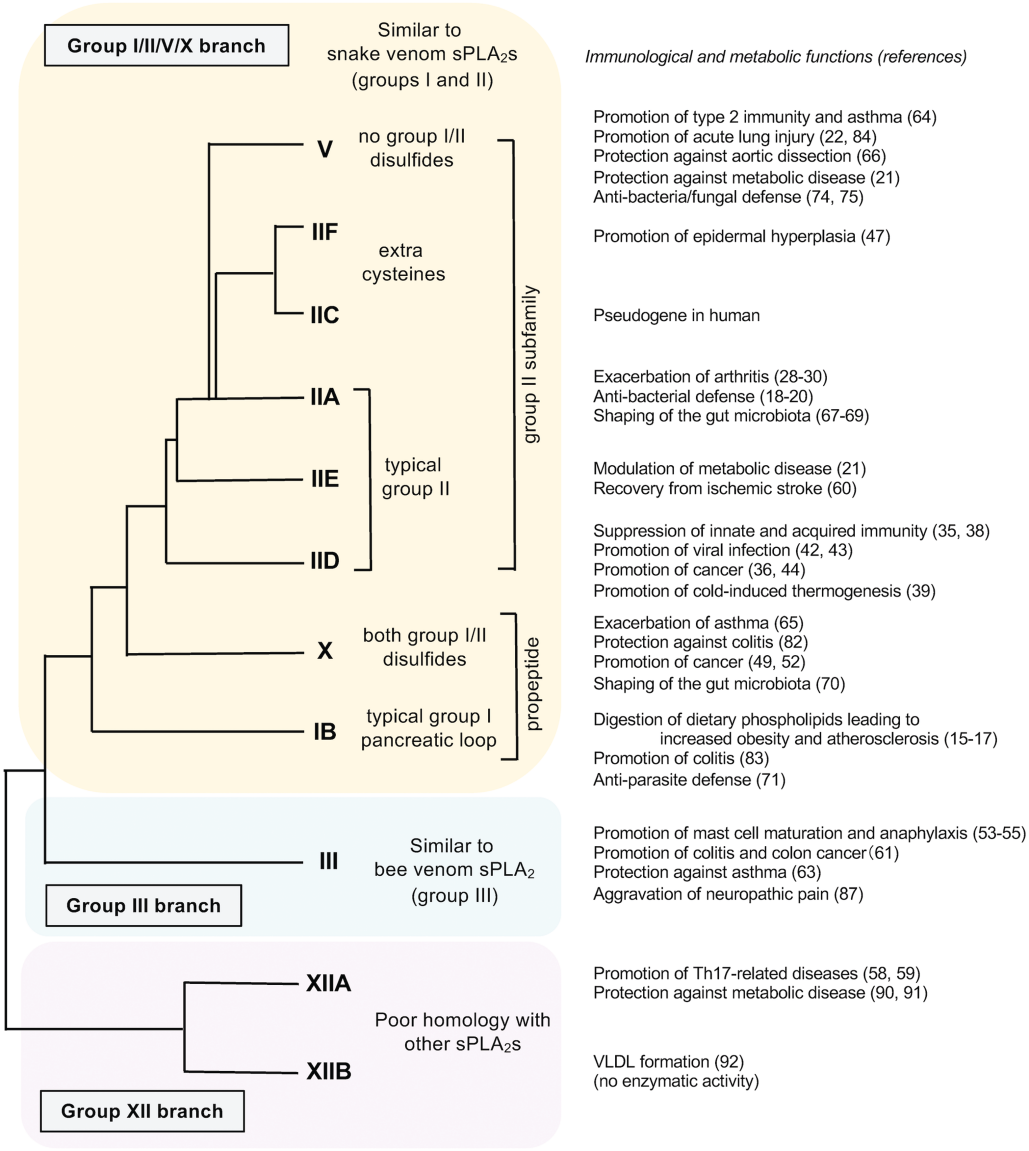
Classification and functions of mammalian sPLA_2_s. Classical sPLA_2_s (group I/II/V/X) are closely related, low-molecular-weight (14−19 kDa) enzymes with a His/Asp catalytic dyad and a highly conserved Ca^2+^-binding loop. They have six to eight conserved disulfide bonds that ensure their high structural stability. The genes for sPLA_2_-IIA, -IIC (present in rodents, but a pseudogene in human), -IID, -IIE, -IIF, and -V are clustered on the same chromosome locus and therefore often referred to as the group II subfamily. sPLA_2_-IB has seven disulfide bonds, including a group I-specific Cys^11^–Cys^77^ disulfide, a unique five amino acid insertion termed the pancreatic loop, and an *N*-terminal propeptide that is proteolytically cleaved for its activation. Typical group II sPLA_2_s (sPLA_2_-IIA, -IID, and -IIE) have seven disulfide bonds, including a group II-specific disulfide linking Cys^50^ with the C-terminal Cys in a C-terminal extension. sPLA_2_-IIC has an extra disulfide bond in a unique sequence in the middle region. sPLA_2_-IIF possesses a long C-terminal extension with a unique free Cys residue. sPLA_2_-V does not have the group I- and group II-specific disulfides and the group II-specific C-terminal extension. sPLA_2_-X possesses both group I- and group II-specific disulfides and an *N*-terminal propeptide. Atypical sPLA_2_s in the group III and XII branches share homology with the classical sPLA_2_s only in the catalytic site and Ca^2+^-binding loop. sPLA_2_-III is an unusually large protein (55 kDa) consisting of a central sPLA_2_ domain with 10 cysteines, flanked with unique *N*- and *C*-terminal domains that are proteolytically removed during protein maturation. sPLA_2_-XIIA and -XIIB (catalytically inactive) are 19-kDa proteins with the lowest homology with other sPLA_2_s. Immunological, metabolic, and other functions of individual sPLA_2_s described in this review are briefly summarized on the right. For details, please see the text. VLDL, very low-density lipoprotein.

The classification and functions of sPLA_2_s are summarized in Fig. 1. In the conventional view, it was speculated that sPLA_2_s act on the outer leaflet of the plasma membrane from outside the cell to release fatty acids and lysophospholipids. However, unlike venom sPLA_2_s that have a potent capacity to cause cell lysis, hydrolysis of membrane phospholipids by mammalian sPLA_2_s hardly occurs in resting cells, except for sPLA_2_-X (encoded by *Pla2g10*), which has the highest affinity for phosphatidylcholine (PC)-rich outer plasma membrane and can release fatty acids even from resting cells when overexpressed or added exogenously at super-physiological levels (11–13). Activated or damaged cells become more susceptible to various sPLA_2_s, likely because phosphatidylethanolamine (PE), phosphatidylserine (PS), and possibly oxidized phospholipids, which have better affinity for most sPLA_2_s, are exposed on the outer plasma membrane (a process called “membrane perturbation”), where sPLA_2_s can augment the production of lipid mediators at least in culture systems (13, 14). However, it remained obscure whether the expression of sPLA_2_s can reach concentrations high enough to hydrolyze cell membrane phospholipids *in vivo*, making it difficult to explain the whole functional aspects of sPLA_2_s expressed in tissues typically at lower levels.

Given the basic property of sPLA_2_ as a secreted protein that requires millimolar order of Ca^2+^ for efficient catalysis, it is reasonable to consider that phospholipids present in extracellular fluids serve as *bona fide* hydrolytic targets of a given sPLA_2_. Indeed, sPLA_2_-IB in pancreatic juice acts as a digestive enzyme to hydrolyze dietary and biliary phospholipids in the gastrointestinal lumen (15–17), sPLA_2_-IIA induced in sera of sepsis patients acts as an antimicrobial protein that destroys bacterial membranes (18–20), and sPLA_2_-V (encoded by *Pla2g5*) hydrolyzes lipoprotein phospholipids during obesity (21) and degrades lung surfactant phospholipids during acute lung injury (22). In addition, phospholipids in EVs have recently emerged as a superior hydrolytic target for sPLA_2_s (23), as described below.

## sPLA_2_ actions on EVs

**Figure 2.**
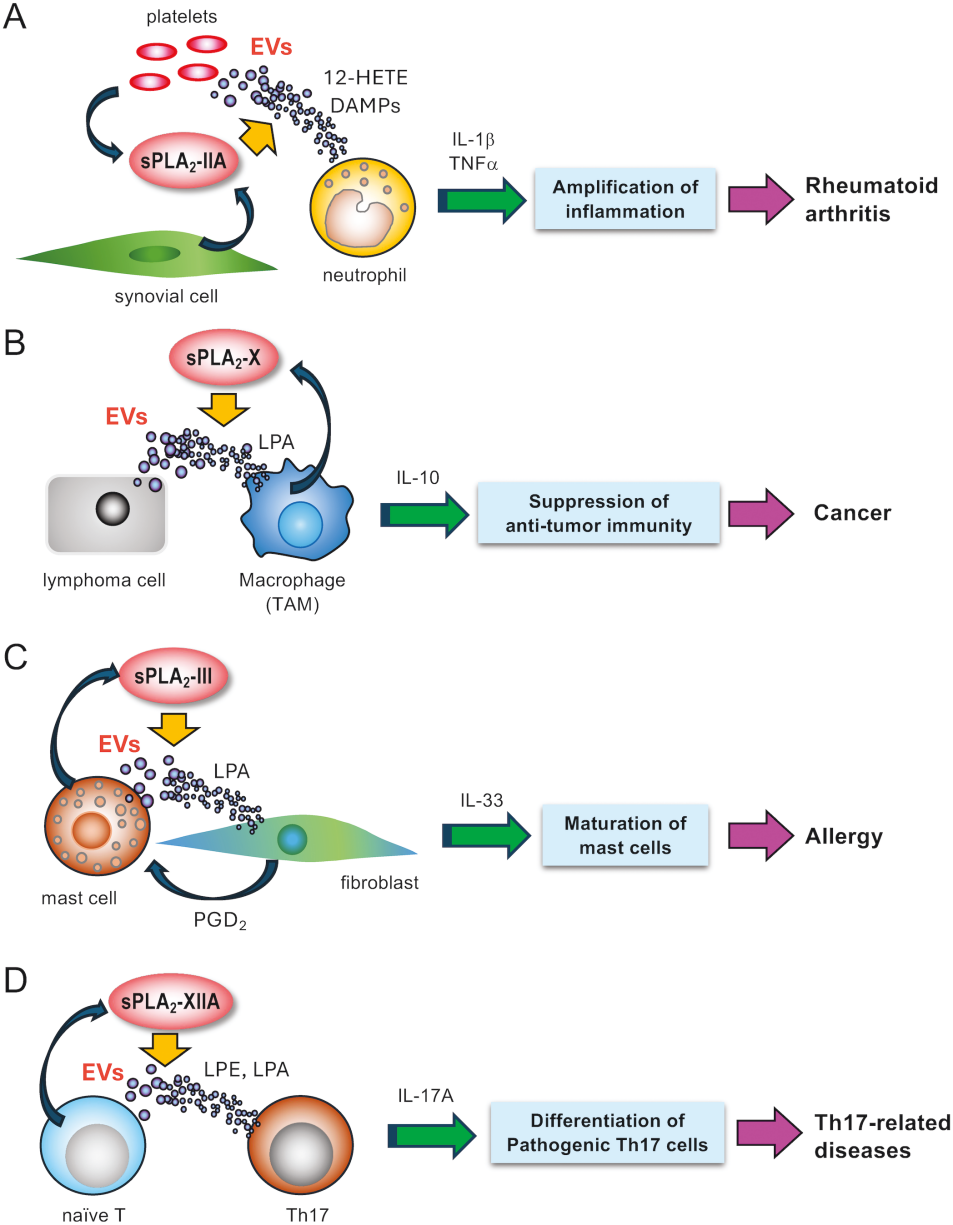
**Paracrine regulation of immunological responses by sPLA**_**2**_ **s via lipolytic modification of EVs**. (A) In rheumatoid arthritis, sPLA_2_-IIA secreted from synovial cells or platelets attacks platelet-derived EVs to generate 12-HETE and DAMPs (e.g. mitochondrial DNA and oxidized lysophospholipids), which activate neutrophils to amplify sterile inflammation. (B) In EBV-induced B-cell lymphoma, sPLA_2_-X supplied by TAMs targets lymphoma-derived EVs to generate LPA, which increases IL-10 secretion by TAMs to exaggerate tumor development. (C) sPLA_2_-III secreted from mast cells hydrolyzes mast cell-derived EVs to generate LPA, which acts on adjacent fibroblasts to increase PGD_2_ and IL-33 that facilitate mast cell maturation. (D) In the context of Th17-driven pathology, sPLA_2_-XIIA secreted from activated T cells hydrolyzes T cell-derived EVs to generate LPE and LPA, which assist the differentiation and expansion of pathogenic Th17 cells. For details, please see the text.

EVs represent small vesicles secreted from cells, such as exosomes (40–150 nm in diameter), which are released via the multivesicular endosomal pathway, microparticles and ectosomes (100–1000 nm), which are produced by plasma membrane budding, and apoptotic bodies (5–10 mm), which are generated from dying cells (24). EVs play crucial roles in intercellular signaling in various stages of cancer, immunity, and metabolism by transferring their cargos (miRNAs, proteins, and metabolites) from donor cells to recipient cells. Although lipids in EVs have long been recognized as merely the “wall” that partitions the lumen of the vesicle from the outside, it has been becoming clear that lipids affect the transfer, uptake and function of EVs, that EVs carry lipid mediators as cargos, and that sPLA_2_-driven hydrolysis of EV membranes modifies their properties and functions (23). Unlike the plasma membrane of resting cells in which PC exists mainly in the outer leaflet and PE and PS in the inner leaflet, EV membranes lose such phospholipid asymmetry, which may account, at least in part, for the reason why EV membranes are more susceptible to most sPLA_2_s. Indeed, sPLA_2_-driven hydrolysis of EV phospholipids does occur in various pathophysiological settings. The actions of sPLA_2_s on EVs are summarized in Fig. 2.

### sPLA_2_-IIA and sterile inflammation

Serum levels of sPLA_2_-IIA are highly correlated with the pathology of various inflammatory diseases, such as sepsis and rheumatoid arthritis (25, 26). In the mid-1990s, Fourcade *et al*. demonstrated for the first time that sPLA_2_-IIA, which is highly induced in synovial fluid of patients with rheumatoid arthritis, could hydrolyze phospholipids in microparticles to produce lysophosphatidic acid (LPA), a pleiotropic lysophospholipid mediator (27). However, this finding did not attract much attention at that time, since the concept of EVs had not yet been established, lipidomics technology did not exist, and the purity of the microparticles used in that analysis was uncertain. About 20 years later, Boilard’s group provided convincing evidence that sPLA_2_-IIA does target large EVs, including microparticles or extracellular mitochondria, derived from platelets and leukocytes (28, 29). The AA released from platelet EVs by sPLA_2_-IIA is converted by 12-lipoxygenase encapsuled in the EVs to 12-hydroxyeicosatetraenoic acid, which promotes the migration and activation of neutrophils via its receptor BLT2 (29). Furthermore, nucleic acids and oxidized lysophospholipids released from synovial EVs by sPLA_2_-IIA activate Toll-like receptors as danger-associated molecular patterns (DAMPs), thus amplifying inflammation (30). These studies have established the concept of sPLA_2_-IIA as a pro-inflammatory factor that aggravates rheumatoid arthritis and likely other sterile inflammation.

Of note, serum levels of sPLA_2_-IIA are 5–10-fold higher in severe coronavirus-infected disease (COVID-19) patients with kidney dysfunction, hypoxia, and multiorgan failure than those in survivors (31). In fact, sPLA_2_-IIA represents a central node in the stratification of patients who died from COVID-19, leading to the proposal that sPLA_2_-IIA is a potential biomarker of COVID-19 mortality. However, it remains unclear whether sPLA_2_-IIA contributes to the “lipid mediator storm” observed in severe COVID-19 patients, and if so, where and how sPLA_2_-IIA mobilizes lipid mediators (from EVs, damaged cells, or else?), which lipid mediators are responsible for the disease pathology, and whether sPLA_2_-IIA inhibition could be effective in treating the disease.

### sPLA_2_-IID and immunosuppression

In the context of metabolic diseases, which have a feature of chronic, low-grade inflammation in metabolically active tissues such as white adipose tissue (WAT) and liver, ω3 PUFAs such as eicosapentaenoic acid (EPA; C20:5) and docosahexaenoic acid (DHA; C22:6) can attenuate inflammation, obesity, and insulin resistance and also increase energy expenditure by facilitating adaptive thermogenesis in brown/beige adipocytes, thereby offering health benefits (32–34). sPLA_2_-IID (encoded by *Pla2g2d*) is abundantly expressed in CD11b^+^CD11c^+^ dendritic cells (DCs) in lymphoid organs and constrains Th1-, Th2-, and Th17-driven immune responses by preferentially releasing ω3 PUFAs (35–37) or possibly by a non-enzymatic mechanism (38). In WAT, sPLA_2_-IID is expressed in M2-like macrophages and inversely correlated with obesity, and global and macrophage-specific sPLA_2_-IID deficiency exacerbates diet-induced obesity and attenuates cold-induced adipocyte browning and thermogenesis (39). Mechanistically, sPLA_2_-IID hydrolyzes adipocyte-derived EVs to preferentially release ω3 PUFAs, which act on the PUFA receptor GPR120 to promote adipocyte browning and thermogenesis, thus counteracting diet-induced obesity, insulin intolerance, and hepatic steatosis (39, 40). Feeding with an ω3 PUFA-rich diet restores the thermogenic capacity and prevents diet-induced obesity in sPLA_2_-IID-deficient mice. These findings underscore the role of the sPLA_2_-IID–EV–ω3 PUFA axis to maintain metabolic health (39) and may be relevant to the finding that a G80S polymorphism of human sPLA_2_-IID is associated with body weight changes in patients with chronic obstructive pulmonary disease (41).

Remarkably, sPLA_2_-IID acts as a critical regulator of antiviral and antitumor immunity through mobilizing immunosuppressive lipid mediators from EVs or through other mechanisms. sPLA_2_-IID-deficient mice are protected against lung injury caused by infection with influenza or coronavirus (CoV), including severe acute respiratory syndrome (SARS)-CoV, Middle East respiratory syndrome -CoV, and pandemic SARS-CoV-2 (42, 43). The absence of sPLA_2_-IID decreases anti-inflammatory PGD_2_ and probably EPA- or DHA-derived oxylipins, thereby augmenting antiviral CD4^+^ and CD8^+^ T cell responses, enhancing pulmonary DC migration into lymph nodes, decreasing lung damage, and increasing survival. In line with this, sPLA_2_-IID-deficient mice are protected from skin cancer because of elevated antitumor immunity with increased CD8^+^ T cells and M1-like macrophages (36). In non-small cell lung cancer, sPLA_2_-IID promotes immune escape by suppressing the functions of CD8^+^ T cells through upregulation of PD-L1 on cancer-derived EVs (44). Moreover, sPLA_2_-IID is a potential prognostic biomarker of various human cancers, strongly correlating with the infiltration of tumor-killing immune cells and the expression of immune checkpoint markers (45, 46). Thus, sPLA_2_-IID may be an attractive drug target for immune checkpoint therapy of viral infection and cancer.

### sPLA_2_-IIF in epidermal-hyperplasic disease

sPLA_2_-IIF (encoded by *Pla2g2f*) is exclusively expressed in epidermal keratinocytes and upregulated in psoriasis and skin cancer (47). sPLA_2_-IIF hydrolyzes keratinocyte-secreted phospholipids, particularly DHA-bearing plasmalogen-type PE, to produce lysoplasmalogen (plasmalogen-type LPE; P-LPE) (47), which augments keratinocyte hyperproliferation through STAT3 phosphorylation (48). This action appears to depend on EV hydrolysis, since sPLA_2_-IIF has the efficient capacity to hydrolyze EVs to preferentially release P-LPE *in vitro* (49). sPLA_2_-IIF deficiency in keratinocytes alleviates psoriasis and skin cancer, accompanied by reduction of skin P-LPE level (47), suggesting that the sPLA_2_-IIF–EV–P-LPE axis is a novel drug target for epidermal-hyperplasic diseases.

### sPLA_2_-X and cancer

Epstein-Barr virus (EBV) infects primate B cells to cause B-cell lymphoma, where virus-derived miRNAs are transferred from lymphoma cells to neighboring macrophages via small EVs (exosomes) to allow skewing of M2-like tumor-associated macrophages (TAMs) that hinder antitumor immunity and thereby promote tumor progression (50). In a model of EBV-infected B-cell lymphoma using humanized mice (immunodeficient NOG mice reconstituted with human hematopoietic cells), sPLA_2_-X is expressed in a population of lipid-laden macrophages and hydrolyzes phospholipids in lymphoma-derived EVs to produce lysophospholipids such as lysophosphatidylcholine (LPC) and lysophosphatidylethanolamine (LPE), which are further converted to LPA by EV-associated autotaxin (ATX), an extracellular lysophospholipase D (49, 51). Then, the LPA-rich EVs elicit strong immunoregulatory signals in recipient macrophages and T cells likely via LPA_1_ receptor. In addition, sPLA_2_-X-treated EVs become smaller, aggregated, and fused, show better uptake, and augment IL-10 expression in TAMs. Furthermore, administration of the pan-sPLA_2_ inhibitor varespladib into humanized mice markedly prevents EBV-induced lymphoma development likely by inhibiting endogenous sPLA_2_-X or other varespladib-sensitive sPLA_2_s, whereas that of sPLA_2_-X-modified EVs facilitates this event (49). These results unveil a novel action mode of sPLA_2_-X in B-cell lymphoma via EVs and open the basis for a novel therapeutic strategy against this malignant disease.

The pro-tumorigenic action of sPLA_2_-X is not limited to EBV-induced lymphoma. A genome-wide screening platform revealed that upregulation of sPLA_2_-X is associated with poor T cell infiltration in various human cancers (52). In a syngeneic tumor graft model, overexpression of sPLA_2_-X in immunogenic tumor cells prevents the infiltration of CD8^+^ T and natural killer cells, thereby conferring resistance to anti-PD-1 immunotherapy. sPLA_2_-X appears to hydrolyze phospholipids to release bioactive lipids, such as LPC that can prevent T cell migration. Inhibition of the enzymatic activity of sPLA_2_-X by varespladib or anti-sPLA_2_-X antibody allows T cell infiltration into the tumor grafts and sensitizes sPLA_2_-X-overexpressing tumors to the immunotherapy. This study provides an additional insight into the pro-tumorigenic role of sPLA_2_-X by dampening T cell immunity and implicates sPLA_2_-X as a potential target for cancer immunotherapy, although the involvement of EV hydrolysis by sPLA_2_-X in this situation remains unclear.

### sPLA_2_-III and allergy

Adherent interaction of mast cells with stromal fibroblasts is essential for their morphological and functional maturation in the tissue microenvironment. Mice deficient in sPLA_2_-III (encoded by *Pla2g3*), the sole mammalian homolog of bee venom sPLA_2_ (group III) known as an anaphylatoxin, are less sensitive to local and systemic anaphylactic reactions because of impaired maturation and activation of mast cells (53, 54). LPA_1_ deficiency in fibroblasts also causes a similar mast cell maturation defect, suggesting the intercellular functional linkage between mast cell sPLA_2_-III and fibroblast LPA_1_ (55). Mechanistically, sPLA_2_-III secreted from mast cells acts on EVs derived from mast cells and/or fibroblasts to produce LPC and LPE, which are further converted to LPA by ATX secreted from fibroblasts. Then, LPA acts on its receptor LPA_1_ on fibroblasts to integrate multiple signaling pathways, including augmented mast cell-fibroblast adhesion by upregulation of the adhesion molecule VCAM-1 on fibroblasts and its integrin ligands ITGB2 and ITGB3 on mast cells, induction of the mast cell maturation cytokine IL-33, increased expression of the PGD_2_ synthase L-PGDS for fibroblastic production of PGD_2_ that acts on the PGD_2_ receptor DP1 on mast cells, and amplification of LPA signaling via upregulation of ATX and LPA_1_ (55). Genetic or pharmacological inhibition of either of these components (sPLA_2_-III, ATX, LPA_1_, VCAM-1, ITGB2/3, IL-33, L-PGDS, or DP1) hampers mast cell maturation and thereby prevents allergic reaction. Moreover, defective mast cell maturation by sPLA_2_-III deletion is fully rescued by supplementation with an LPA_1_ agonist or sPLA_2_-III-modified, LPA-rich EVs, further implying the functional association between sPLA_2_-III and LPA_1_ via EVs. These results reveal a new mechanism of how the paracrine lipid pathway driven by a particular sPLA_2_ controls allergic responses via EV-mediated intercellular communication.

### sPLA_2_-XIIA and Th17 immunity

Metabolic fluxes involving fatty acid biosynthesis play crucial roles in the differentiation of pathogenic Th17 cells (56, 57). A CRISPR-based screening revealed that, in addition to several lipogenic enzymes such as ACC1 (acetyl-CoA carboxylase), FASN (fatty acid synthase), and SCD2 (stearoyl-CoA desaturase), sPLA_2_-XIIA (encoded by *Pla2g12a*), a structurally atypical sPLA_2_ that is secreted from activated T cells and stromal fibroblasts, is involved in pathogenic Th17 differentiation (58). sPLA_2_-XIIA acts on T cell-derived EVs to produce lysophospholipids including 1-oleoyl-LPE, which serves as an activator of RORγt, a signature transcription factor for Th17 differentiation. Furthermore, these lysophospholipids are converted by ATX to LPA, which assists Th17 differentiation mainly via LPA_2_ receptor that augments STAT3 phosphorylation through the G_12/13_-ROCK2 pathway (59). In addition, sPLA_2_-XIIA promotes the secretion and uptake of EVs by Th17 cells and alters their miRNA and protein content. Moreover, defective Th17 differentiation by sPLA_2_-XIIA deficiency is restored by supplementation with an LPA_2_ agonist or sPLA_2_-XIIA-modified EVs. Accordingly, Th17-related disease models such as psoriasis, arthritis, and experimental autoimmune encephalomyelitis are ameliorated by sPLA_2_-XIIA deficiency or anti-sPLA_2_-XIIA antibody treatment (58, 59). These findings represent another example of the sPLA_2_–EV–lysophospholipid axis in fine-tuning immune responses and provide a rationale for targeting the sPLA_2_-XIIA-driven lipid pathway as a potential strategy for the treatment of Th17-related diseases.

## sPLA_2_ actions on damaged cells

As described above, mammalian cells in the resting state are relatively resistant to most sPLA_2_s, and membrane perturbation appears to be required for their hydrolytic action on cell membrane phospholipids. *In vitro*, such membrane perturbation takes place when the cells are strongly activated or undergoing apoptosis or necrosis. Although the *in vivo* relevance of this event had long been obscure, recent studies have revealed that sPLA_2_s indeed appear to act on damaged cells in specific *in vivo* situations, although hydrolysis of EVs (such as apoptotic bodies) might also be involved in this event.

### sPLA_2_-IIE and brain injury

Ischemic brain injury, known as stroke, induces the expression of sPLA_2_-IIE (encoded by *Pla2g2e*) in surviving neurons located at peri-infarct lesions (60). sPLA_2_-IIE releases dihomo-γ-linolenic acid (DGLA; ω6 C20:3) from PS in dying neurons. DGLA is metabolized to 15-hydroxyeicosatrienoic acid (15-HETrE), which promotes neuronal repair for functional recovery. 15-HETrE induces peptidyl arginine deiminase 4 (PADI4), a transcription regulator that upregulates a set of genes associated with the recovery process through histone citrullination, in a subpopulation of surviving neurons. sPLA_2_-IIE deficiency impairs the recovery from ischemic brain injury because of the loss of PADI4-positive neurons, leading to increased inflammation with a reduction of specific neurons that express pro-survival and pro-reparative factors. Moreover, administration of 15-HETrE facilitates functional recovery from ischemic stroke by increasing PADI4-positive reparative neurons. Thus, the sPLA_2_-IIE–DGLA–15-HETrE axis, initiated by sPLA_2_-IIE-driven hydrolysis of damaged cell membranes, underlines a cerebral mechanism for neuronal repair.

### sPLA_2_-III and colonic and lung diseases

sPLA_2_-III is constitutively expressed in colonic epithelial cells, and higher expression of sPLA_2_-III is positively correlated with a higher rate of metastasis and shorter survival in human colorectal cancer (61). sPLA_2_-III-deficient mice show early recovery from dextran sulfate sodium (DSS)-induced colitis and are protected from azoxymethane-induced colon cancer, with reduction of pro-inflammatory and pro-tumorigenic lysophospholipids such as lysophosphatidylinositol and LPA (62). These lysophospholipid changes are observed only after DSS treatment, implying that sPLA_2_-III hydrolyzes damaged membranes in the colon. In a mouse model of antigen-induced asthma, sPLA_2_-III deficiency exacerbates airway hyperresponsiveness, type 2 cytokine expression, eosinophilia, and IgE production (63). In the lung, sPLA_2_-III is constitutively expressed in ciliated, tuft, and neuroendocrine cells of the bronchial epithelium and mobilizes LPC, LPE, and LPA after, rather than before, antigen challenge. Intratracheal injection of LPA_2_ agonists reverses the increased airway hyperresponsiveness and eosinophilia in sPLA_2_-III-null mice, suggesting that sPLA_2_-III constrains allergen-induced asthma by driving tissue-protective epithelial LPA_2_ signaling. In these circumstances, sPLA_2_-III may release lysophospholipids from membranes of damaged cells, EVs, or both. This anti-asthma function of sPLA_2_-III contrasts with the pro-asthma functions of sPLA_2_-V and sPLA_2_-X, which mobilize distinct PUFA metabolites in the lung (64, 65).

### sPLA_2_-V and aortic dissection

sPLA_2_-V is constitutively and abundantly expressed in aortic endothelial cells, being retained on the cell surface by binding to heparan sulfate proteoglycans (66). Upon hypertension induced by angiotensin II (AT-II), sPLA_2_-V plays a protective role against aortic dissection, a life-threatening aortopathy with separation of the aortic wall. Global or endothelial-specific deletion of sPLA_2_-V frequently develops dissection of the thoracic ascending aorta only a few days after AT-II administration (66). In the aorta of AT-II-treated mice, endothelial sPLA_2_-V preferentially mobilizes LA and oleic acid (OA; ω9 C18:1), which attenuate endoplasmic reticulum (ER) stress and increase the aortic expression of lysyl oxidase, thereby stabilizing the extracellular matrix by crosslinking collagens. Moreover, aortic dissection in sPLA_2_-V-deficient mice can be rescued by dietary supplementation with LA and OA. Thus, sPLA_2_-V releases LA and OA from endothelial cells damaged by aortic rupture to maintain aortic stability. This finding may provide a potential mechanism underlying the beneficial effect of OA-rich Mediterranean diet toward cardiovascular health.

## sPLA_2_ actions on microbes

**Figure 3.**
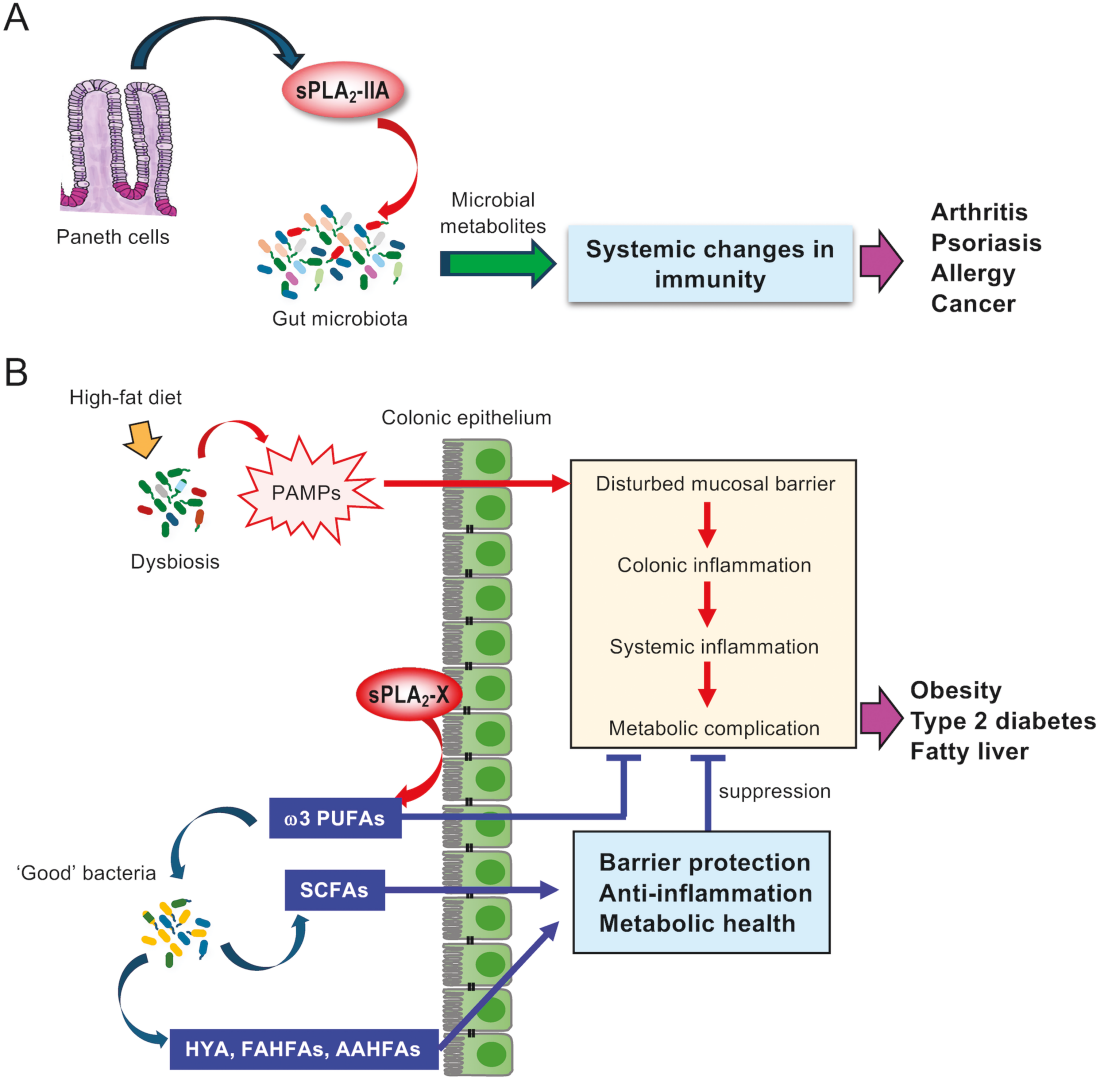
**Regulation of immunological responses by sPLA**_**2**_ **s via modification of the gut microbiota**. (A) sPLA_2_-IIA secreted from intestinal Paneth cells modifies the gut microbiota through its bactericidal activity, thereby offering variable systemic effects. (B) A high-fat diet causes dysbiosis in the gastrointestinal tract, leading to local and systemic inflammation toward metabolic complications. sPLA_2_-X secreted from the colonic epithelium releases ω3 PUFAs from host membranes, which can protect against diet-induced colonic inflammation and increase “good” bacteria. Unique lipids produced by these bacteria, including SCFAs (acetate, propionate, and butylate) as well as LA-derived HYA (10-hydroxy-*cis*-12-octadecenoic acid) and branched-chain fatty acids, including FAHFAs (fatty acid esters of hydroxy fatty acids) and AAHFAs (acyl α-hydroxyl fatty acids), have beneficial effects on systemic immunity and metabolism. For details, please see the text. PAMPs, pattern-associated molecular patterns.

In addition to hydrolysis of endogenous phospholipids in the host membranes, sPLA_2_s are capable of degrading phospholipids in microbial membranes, thereby playing a protective role against infection as antimicrobial proteins. Furthermore, recent studies have revealed an unappreciated role of two intestinal sPLA_2_s, sPLA_2_-IIA in the small intestine and sPLA_2_-X in the colon, in shaping of the gut microbiota with distinct mechanisms (67–70). Importantly, the actions of these sPLA_2_s on the gut microbiota secondarily influence the pathophysiology of distal organs by modulating immunity and metabolism. The actions of sPLA_2_s on the gut microbiota are summarized in Fig. 3.

### sPLA_2_s and host defense

During helminth infection, sPLA_2_-IB secreted from pancreatic acinar cells or a certain population of intestinal epithelial cells acts as an endogenous anthelmintic, eliminating tissue-embedded larvae (e.g. *Nippostrongylus brasiliensis*) by directly degrading worm phospholipids (71). Mice null for sPLA_2_-IB fail to expel the infected worms efficiently, while pretreatment of the larvae with sPLA_2_-IB confers the worm resistance on the host. During bacterial infection, sPLA_2_-IIA induced in the blood and various tissues degrades bacterial membranes, thereby killing Gram-positive bacteria (e.g. *Staphylococcus aureus*), as well as Gram-negative bacteria (e.g. *Escherichia coli*) in the presence of neutrophil-derived antibacterial proteins (18–20). As such, sPLA_2_-IIA-transgenic mice, or wild-type mice treated with recombinant sPLA_2_-IIA, are protected against sepsis or pneumonia following bacterial infection.

Besides the direct action of sPLA_2_s on microbial membranes, they can also contribute to the elimination of microbes indirectly via the hydrolysis of host phospholipids. sPLA_2_-IIA-trangenic mice are resistant to malaria (e.g. *Plasmodium falciparum*) infection, which may involve the action of sPLA_2_-IIA on lipoprotein phospholipids to release oxidized PUFAs that have an inhibitory effect on malaria growth (72). sPLA_2_-V preferentially releases LA from macrophages to allow their skewing into an M2-like phenotype with greater phagocytotic activity, thereby enhancing the phagocytic clearance of harmful microbes (73–75). Accordingly, sPLA_2_-V-deficient mice are more susceptible to *Candida albicans* or *E. coli* infection.

### sPLA_2_-IIA and the gut microbiota

The intestinal tract contains numerous commensal microbes referred to as the gut microbiota, whose composition can give diverse and often profound impacts on the physiological functions of the host (76–78). Unlike in many mammalian species, including humans, sPLA_2_-IIA is expressed only in the intestine of BALB/c and C3H mice and, due to a natural frameshift mutation, it is not expressed at all in C57BL/6 and 129 mice (79). In the small intestine of BALB/c mice, sPLA_2_-IIA expression is limited to Paneth cells, a specific cell type that secretes a cocktail of antibacterial peptides into the intestinal lumen (80). Despite this restricted distribution, sPLA_2_-IIA-deleted BALB/c mice exhibit altered responses to allergy, psoriasis, and cancer in distal skin compared to wild-type mice (68, 69). These phenotypes in sPLA_2_-IIA-deficient BALB/c mice are abolished when the mice are housed in a cleaner facility, treated with antibiotics, or cohoused with wild-type mice, suggesting the contribution of the gut microbiota. Indeed, metagenomic and metabolome analyses revealed that some fecal bacteria (e.g. *Lachnospiraceae*, *Ruminococcaceae*, and *Helicobacteraceae*) are changed in sPLA_2_-IIA-deficient BALB/c mice, accompanied by altered blood levels of several water-soluble metabolites as well as decreased fecal levels of bacterial lipids such as unique oxygenated LA metabolites and branched fatty acid esters that have immunoregulatory properties (68, 69). Moreover, transgenic overexpression of sPLA_2_-IIA in C57BL/6 mice also alters fecal abundance of Gram-positive bacteria, which is associated with exacerbation of arthritis and splenomegaly (67). Thus, bactericidal sPLA_2_-IIA secreted into the intestinal lumen affects the pathology of distal tissues by shaping of the gut microbiota through degradation of bacterial phospholipids.

### sPLA_2_-X and the gut microbiota

sPLA_2_-X is highly expressed in epithelial cells of the stomach and colon but is barely detectable in metabolic and immune tissues (70, 81, 82). Despite this biased distribution, mice deficient in sPLA_2_-X display obesity-related phenotypes that are lost after treatment with antibiotics or cohousing with wild-type mice, suggesting the contribution of the gut microbiota (70). Colonic sPLA_2_-X hydrolyzes host membranes to release ω3 PUFAs, which counteract diet-induced dysbiosis, colonic inflammation, and barrier dysfunction. Furthermore, the release of ω3 PUFAs by sPLA_2_-X leads to an increase of fiber-degrading *Clostridium,* a bacterial species that produces short-chain fatty acids (SCFAs) with beneficial effects on host metabolism and immunity. Dietary supplementation with ω3 PUFAs or SCFAs rescues the obesity-related phenotypes in sPLA_2_-X-deficient mice (70). Thus, colonic sPLA_2_-X orchestrates the ω3 PUFA–SCFA interplay via shaping of the gut microbiota, thereby secondarily affecting systemic metabolism. It remains to be elucidated whether sPLA_2_-X releases ω3 PUFAs from cell membranes or EVs in this situation, whether sPLA_2_-X directly kills certain bacteria through its intrinsic bactericidal activity (as in the case of sPLA_2_-IIA), or whether some other bioactive lipids mobilized by sPLA_2_-X could also have impacts on the microbiota.

## Other functions of sPLA_2_s with ambiguous target substrates and products

In addition to the functions described above, several immunological and other roles of sPLA_2_s have been reported in the past few years, although the underlying lipid metabolism (target membranes and key metabolites) is less clear. sPLA_2_-IB deficiency enhances disease recovery from DSS-induced colitis (83), which may be related to the role of this enzyme in phospholipid digestion in the intestinal lumen and associated changes in the gut microbiota. Lung inflammation induced by polyIC administration (mimicking viral infection) is reduced in sPLA_2_-V-deficient mice, probably because of an alteration in the macrophage lipidome (84). On the contrary, sPLA_2_-IIE-deficient mice are more sensitive to influenza infection with reduction of T cell immunity, where an alteration in the T cell lipidome might be involved (85). In a meningitis model caused by *Streptococcus suis* infection, disruption of the blood-brain barrier by a bacterial toxin depends on sPLA_2_-III induced in endothelial cells (86). In a model of neuropathic pain, hyperalgesia and hypoalgesia caused by sciatic nerve ligation are reduced by sPLA_2_-III silencing (87). Likewise, in a model of temporomandibular disorders, malocclusion-induced orofacial and somatic hyperalgesia is attenuated by an anti-sPLA_2_-III antibody (88). However, the lipid metabolism underlying the sPLA_2_-III actions in pain sensation is unclear.

Beyond the scope of this review, the biological effects of sPLA_2_s may also be driven or counter-regulated by binding to membrane-bound and soluble forms of sPLA_2_ receptor (PLA2R1) or other sPLA_2_-binding proteins (8). Intestinal PLA2R1 may transmit sPLA_2_-IIA and sPLA_2_-X signals to regulate gut homeostasis (80), while pulmonary PLA2R1 may play a role in the clearance of pro-asthmatic sPLA_2_-X (89). sPLA_2_-XIIA has a protective role against obesity and insulin resistance possibly in a catalytic activity-independent manner (90, 91), although the mechanistic action of this atypical sPLA_2_ needs further elucidation. sPLA_2_-XIIB (encoded by *Pla2g12b*), a catalytically inactive sPLA_2_ isoform, regulates lipoprotein biogenesis in the liver by acting as an ER-resident lipid chaperone (92).

## Conclusions

Recent findings summarized in this review have provided new insights into the action modes of sPLA_2_s. First, EV phospholipids serve as excellent hydrolytic targets for various sPLA_2_s in immunity and cancer. Second, sPLA_2_s act on membranes of damaged cells rather than live cells in specific *in vivo* circumstances. Third, intestinal sPLA_2_s affect distal organs indirectly through shaping of the gut microbiota. These mechanisms could explain many, even if not all, of the *in vivo* functions of sPLA_2_s, although generalization of this concept needs further investigations in the future. From the perspective of immunology and lipid biology, sPLA_2_s can be regarded as lipolytic cytokines, a distinguished class of immune regulators that influence the functions of various immune and non-immune cells in a paracrine manner via mobilization of bioactive lipids from extracellular phospholipids. From the perspective of EV biology and microbiology, sPLA_2_s can be regarded as a novel class of modifiers of EVs and the gut microbiota. As such, sPLA_2_ research will offer widespread impacts on a broad range of life science. Nonetheless, since many of the functions of sPLA_2_s have been based on studies using mouse models, it is important to translate these findings to humans. Hopefully, a more comprehensive picture of the sPLA_2_-driven lipid networks will be demonstrated during the next decade, thus allowing the opportunity for therapeutic application of sPLA_2_-targeted inhibitors or antibodies to human diseases.
